# An adaptive power system transient stability assessment method based on shared feature extraction

**DOI:** 10.1016/j.isci.2025.112172

**Published:** 2025-03-06

**Authors:** Jiexiang Hu, Le Zheng, Wei Ai, Yansong Li, Jun Liu, Xinglei Chen

**Affiliations:** 1 Changsha Power Supply Branch, State Grid Hunan Electric Power Co., Ltd., Changsha 410035, China; 2 Department of Electrical and Electronic Engineering, North China Electric Power University, Beijing 102206, China; 3 School for Environment and Sustainability, University of Michigan, Ann Arbor, MI 48109, USA; 4 China Electric Power Research Institute, Haidian District, Beijing 100192, China

**Keywords:** Engineering, Energy systems

## Abstract

Machine learning-based power system transient stability assessment (TSA) faces challenges with performance degradation under varying operating scenarios. This paper proposes a robust and transferable adaptive TSA method based on shared feature extraction of the power system. A domain adversarial alignment network is used to train a shared feature extractor, aligning data before and after system variations to capture critical stability features. This reduces the need for extensive labeled data and improves assessment across different scenarios. When the system scenario changes, data and model knowledge are transferred simultaneously, maintaining high accuracy even with significant data loss in new scenarios. Testing on the IEEE 39-bus system and a 2179-node province-level system shows that the method achieves over 96% prediction accuracy with 30% data loss and sustains 97.99% accuracy in continuously changing scenarios, outperforming traditional methods. The results demonstrate the method’s potential for real-world application with enhanced generalizability, robustness, and sustainable learning capability.

## Highlights

• Designed a shared feature extractor for invariant transient stability features
• Measured data disparity before and after scenario change to guide data alignment
• Validated adaptive TSA framework on IEEE 39-node and 2179-node power systems in China
• Enhanced TSA model robustness; target domain accuracy >96% even with 30% data loss

## Graphical abstract

**Figure:**
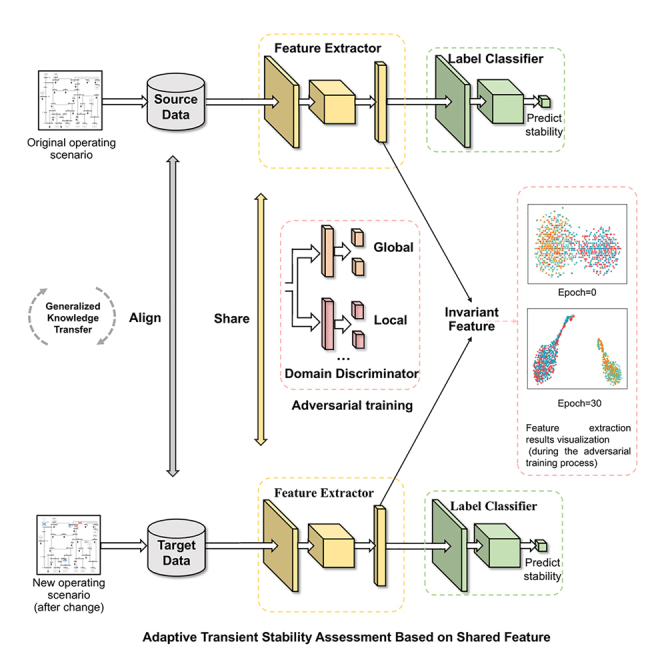

## Introduction

The scale and complexity of the power system are growing with the energy transition, introducing significant challenges to the safe and stable operation of the power system. Therefore, a fast and reliable transient stability assessment (TSA) is crucial for the modern power system. Traditional TSA methods include the energy function-based direct method^1^^,^^2^^,^^3^ and the time-domain simulation method.^4^^,^^5^^,^^6^ While the direct method can quickly provide TSA through the energy function, it tends to produce large calculation errors when applied to complex, large power grids.^1^^,^^2^ The time-domain simulation method can provide accurate results, but its computation is slow, and its hardware requirements are high.^5^ As a result, both methods face challenges in meeting the demands of modern, large power grids.

Luckily, the widespread development of grid-wide area measurement systems, combined with the advanced data mining capabilities of machine learning (ML), is fueling a significant increase in the application of ML techniques in TSA. The basic structure of the ML method in TSA applications includes three steps: (1) collect historical and simulation data to create sample sets for power system TSA; (2) use the sample sets to train TSA models; and (3) input the online operation data of the power system to the trained model to conduct TSA. In Yu et al.’s study,^7^ the training data were generated using time-series simulation of various transient contingencies on the given power system. The TSA model based on long short-term memory (LSTM) was trained offline and then applied online. Similarly, Wu et al.^8^ employed a deep belief network architecture, which offers higher interpretability of the relationship between system features and the assessment results. In another studied, Li et al.^9^ designed a TSA model based on convolution algorithms and deep forest and tested the model’s performance on the IEEE 39-node and 500-node systems.

TSA models established based on the aforementioned structure implicitly assume that the dataset used for model training follows the same distribution as the actual measurement data in the power system. However, this assumption is not always valid. The operating scenarios of the power system may undergo unexpected changes, and different operating scenarios result in different distributions of power system data. Since the training dataset cannot cover all possible variations in operating scenarios, the performance of the trained TSA model inevitably declines under unseen operating scenarios. This significantly affects the adaptability of the trained TSA models. A simple solution to this problem is to retrain the TSA model using newly labeled datasets when the system’s operating scenario changes. However, it requires a substantial amount of time and expertise, which does not accommodate to practical applications.

Transfer learning is an effective technique to address the aforementioned issue. It manages to make the best use of the similarity between the source (original operational scenarios) and the target (new operational scenarios) domain TSA prediction task. To be specific, the original TSA model is updated in some way when the operating scenario changes. Currently, transfer learning-based TSA can be classified into three categories: sample transfer,^10^^,^^11^ model transfer,^12^^,^^13^ and feature transfer.^14^^,^^15^^,^^16^^,^^17^^,^^18^^,^^19^

(1) Sample transfer methods involve selectively filtering samples from labeled source domain data to aid in the target domain training. Li et al.^10^ used vector distance to assess the similarity between typical samples from the source and target domains, facilitating the selection and transfer of source domain samples. Shi et al.^11^ employed active learning to identify samples with valuable labels while discarding less useful ones from the original dataset, creating a hybrid dataset to enhance transfer learning in the target system. The effectiveness of sample transfer methods heavily relies on the subset selection from the source domain dataset that sufficiently represents the samples in original scenarios and maximizes similarity with the target domain dataset. However, this process often requires significant expertise or involves complex and time-consuming training, results in updating challenges.
(2) Model transfer methods apply a pre-trained model from the source domain after fine-tuning it using labeled data from the target domain. Maeshal et al.^12^ fine-tuned the parameters of the basic model using labeled data from the new topology. Todeschini et al.^13^ froze the convolutional layers and fine-tuned the unfrozen layers to suit the new task of classifying voltage waveforms. Zhang et al.^20^ designed retraining strategy by determining the frozen layer and fine-tuning The training process was simplified and high predictive performance was achieved. However, model transfer methods update the training model with new tasks, requiring many labeled samples in the target domain and potentially losing valuable information in the existing model. The fine-tuned model may struggle to maintain accurate assessment in the original operational scenario and experiences “catastrophic forgetting”. In practical applications, the operational scenarios may continuously change and even fluctuate repeatedly. For instance, generators or industrial plants could be switched on or off. Therefore, it is crucial to update TSA models amid evolving operational scenarios while retaining the knowledge that has already been gained.
(3) Feature transfer methods consider the knowledge from both, the source and the target domain, necessitating fewer labeled data and having lower requirements for data distribution differences in the target domain than other methods. To be specific, a feature transformation is optimized to minimize the distribution discrepancy between the original and new operational scenarios, thereby aligning the data from new scenarios with those from the original scenarios. In Ren and Xu’s study,^14^ the source and target domains are mapped to a regenerated reproducing kernel Hilbert space with high similarity. The distance between data is measured based on the maximum mean discrepancy (MMD), and the data distribution difference is iteratively minimized to improve the performance of the trained TSA model in the target domain. Ren et al.^14^ performed dimensionality reduction on the data and used MMD to measure the distribution of target and source domain data. Besides, other metrics like Euclidean distance,^21^ cosine similarity,^22^ and Kullback-Leibler divergence^23^ can also be used to measure the data distance. However, these predefined metrics often come with limitations to adequate characterize differences in dynamically changing data distributions; for instance, cosine similarity disregards data magnitude, MMD exhibits reduced accuracy when applied to large, high-dimensional datasets, etc. Consequently, researchers have endeavored to formalize a learnable metric, enabling models to autonomously acquire this metric from data. Ma et al.^17^ employed principal component analysis and density-based spatial clustering of applications with noise to identify common features with minimal distribution distances. Zhao et al.^18^ utilized manifold embedded distribution alignment to transform features from their original space to a manifold space, achieving beneficial geometric properties and reducing data drift between the source and target domains for fault diagnosis based on limited labeled data in the target domain. However, these feature transfer methods require an initial analysis of the data distribution and the measurement of distribution differences between the source and target domains, along with the resolution of data alignment. After this, the source domain data, which has undergone alignment transformation, is fed into the TSA model for transient stability feature extraction and label classification, which is used to train the TSA model, ensuring it provides reliable performance for assessment. Finally, the well-trained model is applied to perform TSA on the target domain data. In other words, beyond model training and application, analyzing and aligning the data distributions are required. This additional process involves complex calculations and time-consuming neural network training, making the transfer process more intricate. This requirement can be avoided by using the feature extractor within the TSA model for data feature transformation. According to the principle of power system dynamics, similar patterns exist in TSA data, such as voltage drops and divergence in the power angles of system generators post faults. The data alignment method in feature transfer makes the most use of these patterns. In this way, the generalizability of TSA models, which is interpreted as in the following, is improved. First, one TSA model can be utilized in both original and new operating scenarios. Second, data in other scenario may be included within the aligned domains. However, this generalizability has not yet received widespread attention and application. Moreover, considering power system operating scenarios change randomly, the degree and mode of data variations differ among different system scenarios (e.g., generator withdrawal, line faults, and generator output fluctuations). Consequently, the disparity between data in different operating scenarios vary, which has rarely been considered in previous studies. In the field of machine learning, Yu et al.^19^ demonstrated a mismatch between global distribution and local distribution during data changes, showing that addressing distribution mismatches dynamically can improve model test results from around 77% to about 91% under identical scenarios and accelerate model convergence. Hence, it is essential to dynamically track data changes following power system operational shifts and effectively align transient stability feature across different operating scenarios.

In response to these issues, a robust and transferable adaptive TSA method based on shared feature extraction is proposed in this paper. By analyzing the characteristics of power system data, a feature extraction network for grid data is constructed. When the power grid operating scenario changes, both the data and model knowledge from the original scenario are transferred. A substantial amount of labeled data from the original scenario is utilized to assist in training the TSA model for the new scenario. The distribution differences of sample data before and after the change in operating scenarios are adaptively measured. Through adversarial training, the data under the two operating scenarios are aligned, while simultaneously enhancing the accuracy of the TSA model. The loss of the domain discriminators and label classifier are computed, and the TSA model is updated through backpropagation. Ultimately, a shared feature extractor and the corresponding label classifier, applicable to both operating scenarios, are obtained, enabling reliable TSA under the new operating scenario. The main contributions are as follows.

(1) A shared feature extractor with high generalizability was designed to extract transient stability features that remain invariant despite changes in power grid operating scenarios. It simultaneously performs feature extraction and aligns data from both the original and new operating scenarios. Its effectiveness was visualized using the t-distributed stochastic neighbor embedding (t-SNE) technique, and its resilience in handling data loss was also demonstrated.
(2) The disparity of data before and after operation scenario change is adaptively measured, including both local and global distribution differences, which guides data alignment.
(3) An adaptive TSA framework was constructed, which simultaneously transfers both the data and model knowledge from the original operating scenario when the power grid operating scenarios change. It was verified on the IEEE 39-node system and a 2179-node system in Northeast China, demonstrating its generalizability, robustness, and sustainable learning capability.

## Results and discussion

Major case studies are performed on the IEEE 10-machine 39-node power system^24^ to demonstrate the advantages of the proposed TSA method. Additionally, a large system test is carried out on a provincial-level power system with 2,179 nodes in Northeast China to highlight the method’s viability for real-world large power systems. The TSA datasets are generated through transient simulations using the Power System Analysis Software Package (PSASP) v7.51. The deep learning module is implemented using PyTorch 1.8.1. All the tests are performed on a personal laptop (CPU: AMD Ryzen 7 5800H with Radeon Graphics and 16 GB of RAM, GPU: NVDIA GeForce RTX 3060 Laptop).

To represent possible changes in real power system operating scenarios, three target power systems^25^^,^^26^^,^^27^ were created based on the IEEE 39-nodes standard system^28^^,^^29^^,^^30^^,^^31^: Target system 1 representing partial generator tripping and output changes, target system 2 representing generator output fluctuations, and target system 3 representing topological changes caused by line faults. The system setups are explained in the following text:

Source system S0: For the IEEE 39-node standard system, load levels are set to increase in increments of 5% between 80% and 120%, resulting in nine different load conditions. For each load condition, two severe faults are introduced at the 2%, 25%, 50%, 75%, and 98% positions of all lines: three-phase short circuit faults and single-phase short circuit faults. The faults start at 1.0 s and last for 0.1 s–0.3 s, increasing in increments of 0.05 s. Successful simulations yield 14,243 transient stable samples, including 9,391 stable samples and 4,852 unstable samples.

Target system T1: One generator (G1) and one line (BUS30-BUS2) are disconnected from the IEEE 39-node standard system. Load levels are set to increase in increments of 5% between 80% and 120%, resulting in nine different load conditions. The same fault setting method as the source domain system is used. Successful simulations yield 14,251 samples, including 7,757 stable samples and 6,494 unstable samples.

Target system T2: The system’s load level is adjusted to 130% of the standard load, and the generator’s output level is adjusted to 105% of the standard output. The system’s topology remains unchanged. The same fault setting method as the source domain system is used. Successful simulations yield 1,600 samples, including 1,056 stable samples and 544 unstable samples.

Target system T3: Two lines (BUS26-BUS27 and BUS27-BUS28) are disconnected from the system. Load levels are set to increase in increments of 5% between 80% and 120%, resulting in nine different load conditions. The same fault setting method as the source domain system is used. Successful simulations yield 13,012 samples, including 7,131 stable samples and 5,881 unstable samples.

### Performance testing of shared feature extractor

**Figure 1:**
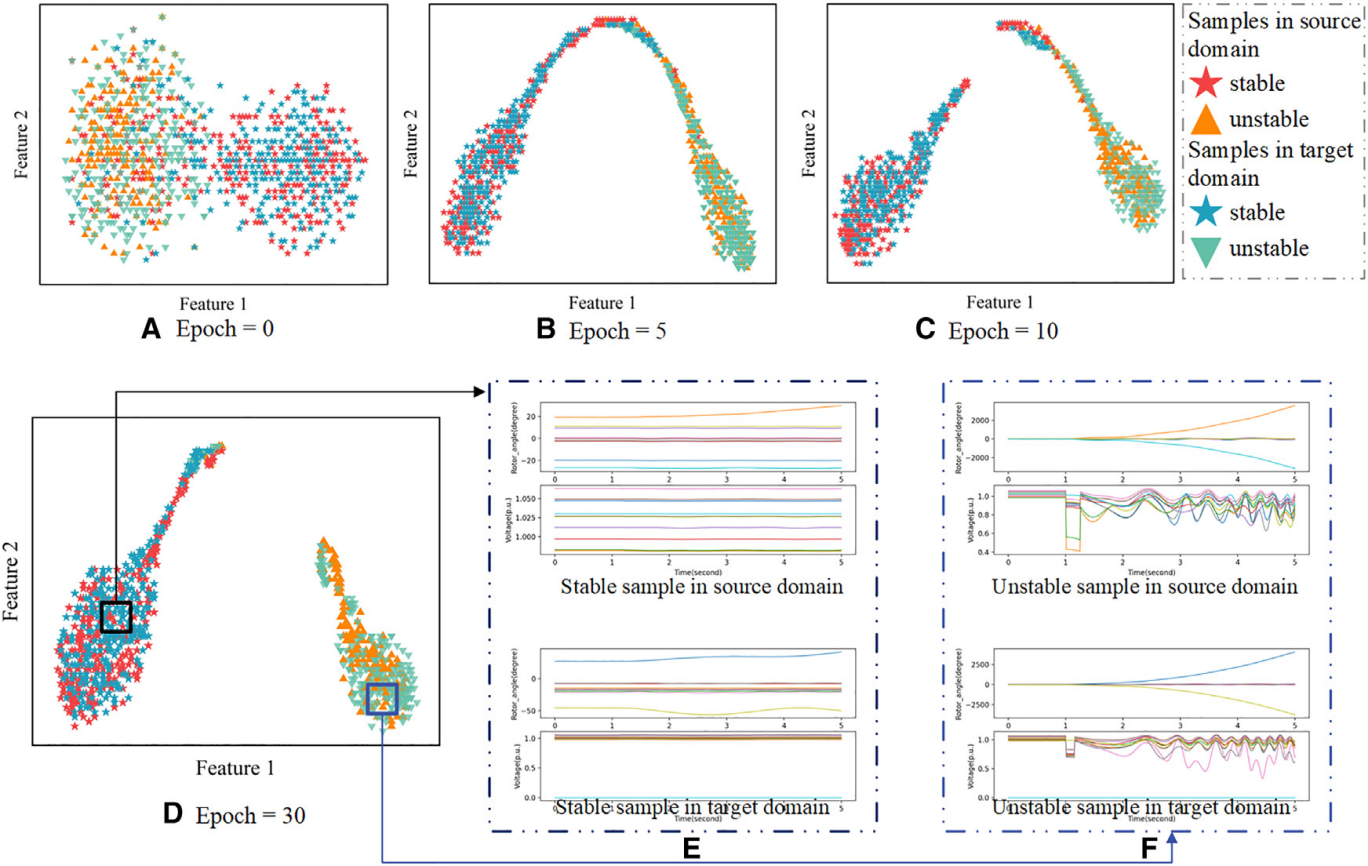
Evolution of feature distribution through adversarial domain adaptation (A–D) Visualization of the feature space during training: (A) initial distribution at epoch 0 showing distinct clusters of source and target domains; (B) intermediate alignment at epoch 5 demonstrating improved feature overlap; (C) progressive domain convergence at epoch 10; (D) feature distribution at epoch 30 showing significant domain alignment with two highlighted regions. (E and F) The panels compare trajectory for: (E) stable samples showing consistent convergence patterns across source and target domains; (F) unstable samples exhibiting characteristic oscillations in both domains. Color coding indicates sample stability (stable/unstable) and domain origin (source/target) as shown in the legend.

During the adversarial training process, the feature extractor is alternately trained. Initially, the loss of transient stability state classification is minimized, enabling the feature extractor to effectively capture transient stability features of the power grid. Based on the extracted transient stability features, reliable TSA is performed. Then, the loss of global domain discriminator and local domain discriminator under both the original and new operating scenarios is maximized, aligning the global and local distributions of the source and target domain data. Through backpropagation, the feature extractor is updated to achieve both data alignment and TSA feature extraction. The extracted high-dimensional features of the power system are visualized through dimensionality reduction. Figure 1 shows the feature extraction results during the adversarial training process. It can be observed that the feature distributions gradually become more similar and the initial significant differences are reduced. The obtained shared feature extractor effectively distinguishes stable and unstable samples from both the source and target domains, verifying the model’s successful extraction of stability-related characteristics. Analyzing the overlapping sample data from the source and target domains in the figure reveals that data with the same features exhibit similar electrical patterns, particularly in terms of voltage and rotor angle fluctuations after system faults. This further verifies the effectiveness of the shared feature extractor in extracting electrical information related to transient stability.

**Figure 2:**
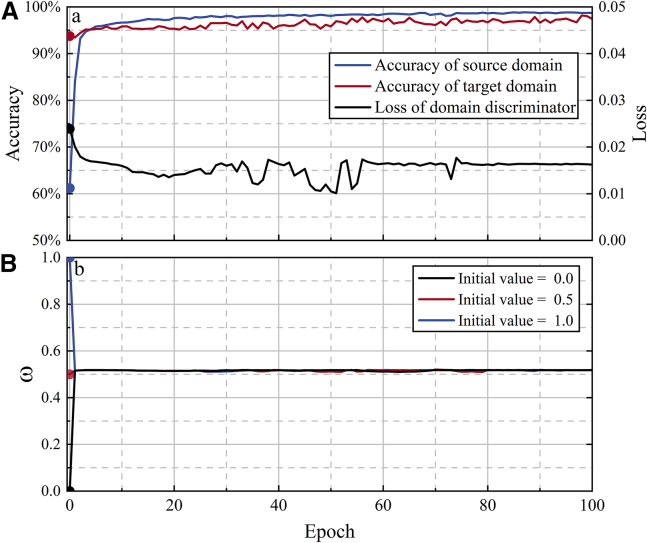
Training dynamics over iterations (A) Concurrent evolution of classification accuracy and discriminator loss over training epochs, showing high source domain accuracy (blue) and target domain accuracy (red) alongside diminishing domain discriminator loss (black). The dual y axes represent accuracy (left) and loss values (right). (B) Convergence behavior of the dynamic adversarial factor (ω) over 100 epochs under different initialization values (0, 0.5, and 1), demonstrating robust convergence to an optimal value (∼0.5) regardless of starting conditions.

The variations in loss and accuracy during the training process are shown in Figure 2A. The objective of adversarial training is to simultaneously achieve alignment between source and target domain data and reliable TSA. A larger domain classification loss (including global and local domain adversarial losses) indicates a smaller feature distribution gap and makes it easier to “confuse” the domain classifier. The domain classification loss shows a decreasing trend and eventually stabilizes at 0.017. This suggests that during the iterative adversarial training, the high-dimensional features extracted tend to be similar between the source and target domains, and the prediction accuracy for both domains improve. Moreover, the proposed method uses a shared extractor and label classifier to conduct TSA for both source and target domains, resulting in the model that can demonstrate good applicability to the system both before and after operational scenario changes. After the first iteration, the prediction accuracy in the target domain increases to 93.75%, and the prediction accuracy in the source domain increases from 61.20% (prior to training) to 85.98%. After 100 training epochs, the prediction accuracy in both domains reaches 98.73% and 98.18%, respectively.

The dynamic adversarial factor *ω* quantifies the differences in the global and local distributions by utilizing the losses from the global domain discriminator and two types of local domain discriminators, each corresponding to stable and unstable cases, respectively. This enables adaptive fine-tuning without the need for additional classifier construction to compute distances, facilitating rapid adaptation to power system operation changes and grid data distribution disparities. Three different initial values (0, 0.5, and 1) are assigned to the dynamic adversarial factor *ω* to test the adaptive measurement capability, as shown in Figure 2B. Under various initial values, the dynamic adversarial factor *ω* converges adaptively to the same measurement factor within three iterations, validating the effectiveness of using the dynamic adversarial factor *ω* to measure the global and local distribution disparities between the source and target domains.

During the real-time operation of the power system, accumulation of transient stability data in new scenarios is insufficient. Achieving optimal assessment results with the minimum number of samples has attracted the attention of operation and dispatch personnel. Based on basic structure (as mentioned in the 2^nd^ paragraph of introduction), the performance of a model approaches its limit as the accumulation of target domain samples increases. The proposed adaptive updating method based on shared feature extraction enables transferring both model and data knowledge, improving the utilization of source domain knowledge. The retraining method, fine-tuning, Kernel mean matching (KMM), and transfer component analysis (TCA) are selected for comparison with the proposed method. After power system operating scenarios change, the required sample quantities for these methods to achieve the same accuracy (96%) in the target system (T1) are tested. The tests are conducted with a step size of 5% of the sample quantity. The results are shown in Table 1. The test results demonstrate that, among the tested models, the proposed method requires the fewest target domain samples for updating and can meet the requirements of online scheduling.

**Table 1:** Comparison of different transfer learning method (ACC 96%)

Method	Retain	Fine-tune	KMM^a^	TCA^b^	Proposed method
Labeled Data Needed	30%	15%	10%	10%	5%

a Kernel mean matching.

b Transfer component analysis.

Feature extractor performance is assessed while keeping the label classifier structure unchanged. The (convolutional neural network) CNN, LSTM, and support vector machine (SVM) models, widely used in TSA, are selected for comparison. These four models are trained and validated using the source domain data, and their predictive performance is evaluated on the test set. The CNN and LSTM algorithms are trained with the following parameters: a learning rate of 10^−3^, a minimum learning rate of 10^−8^, and a cosine annealing strategy for learning rate decay. Batch sizes of 128 and 100 iterations are used. For SVM, the shallow learning algorithms, principal-component analysis (PCA) is employed to reduce the dimensionality of the original data, serving as input for shallow learning (SVM). The comparative performance of the methods in TSA is presented in Table 2. The test results clearly indicate that the deep learning (CNN and LSTM) models outperform the shallow machine learning models in terms of accuracy, missing rate, and false alarm rate for transient stability prediction. Among these models, the feature extractor architecture proposed in this paper achieves the highest accuracy of 99.35%. It also has lower M_is_ and F_al_ rates than the CNN and LSTM models overall. This is because the feature extractor proposed in this paper adapts to the spatiotemporal characteristics of power system data—specifically the correlation between electrical quantities and the time-varying nature of measurements—enabling more effective extraction of features from transient stability data.

**Table 2:** TSA model performance of different neural networks

Model	Evaluation metrics		
	Accuracy/%	Missing rate/%	False alarm rate/%
This study	99.35	1.20	0.43
CNN^a^	98.98	0.81	1.13
LSTM^b^	98.84	1.01	1.24
SVM^c^	97.57	2.52	2.37

a Convolutional neural network.

b Long short-term memory.

c Support vector machine.

### Interpretability analysis of TSA model before and after adversarial training update

**Figure 3:**
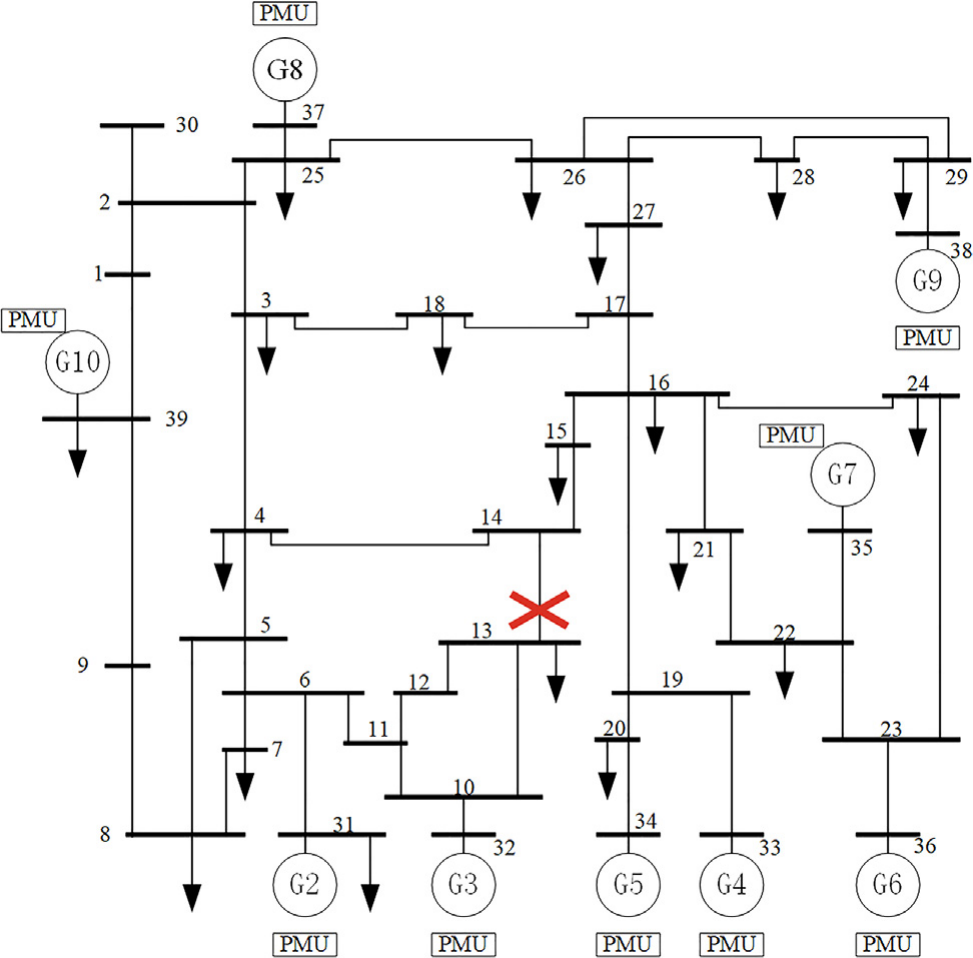
IEEE39 node system single line diagram and fault location

**Figure 4:**
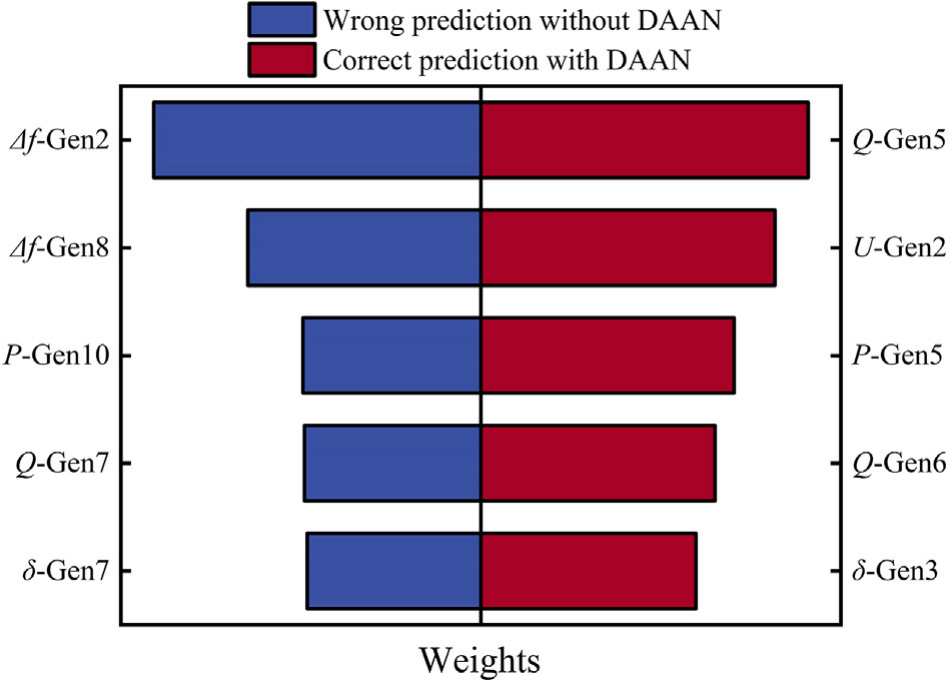
Feature importance rankings and weights before and after adversarial training

Regarding a specific fault in the target domain T1 (the sample to be explained, determined as an unstable sample through simulation), as depicted in Figure 3, we assess the transient stability using both the pre- and post-updated models. The local interpretable model-agnostic explanations (LIME) algorithm^32^ is employed to interpret the assessment results of these models, by analyzing the coefficients or feature importance of the interpretable model. The outcomes of the top five weighted features are presented in Figure 4, where the weight’s magnitude reflects the importance of the feature in predicting. The positive or negative sign indicates the direction of the feature’s impact on accurate predictions.

As shown in Figure 4, after adversarial update training, the TSA model’s stability assessment of the sample changes from incorrect to correct, and the influence direction of the top five ranked features on the model’s prediction also changes from incorrect to correct. Further analysis is conducted on the generators corresponding to the top five weighted features. As can be seen from the single-line diagram of the system (Figure 3), generators G2, G3, and G5 are the closest to the fault location, and they are the ones most affected by the fault, significantly impacting system instability. Among the top five weighted features of the updated TSA model, four correspond to electrical quantities of generators G2, G3, and G5. In contrast, the top five weighted features of the pre-updated TSA model correspond to geographically dispersed generators, with only one of these generators located near the fault. The test results indicate that adversarial training effectively updates the model’s feature extraction and prediction capabilities.

### Sustainable learning capability, generalizability, and robustness test

In practical applications, the operational scenarios may continuously change and even fluctuate repeatedly. For instance, generators or industrial plants could be switched on or off, load may fluctuate, and system may recover after fault removal. In this paper, when the operating scenarios change, adversarial training is utilized to obtain a shared feature extractor that considers both the source and target domain knowledge, maximizing the knowledge capacity of deep neural network models. As a result, the proposed method effectively avoids the “catastrophic” forgetting problem and demonstrates excellent sustainable learning ability in continuously changing operating scenarios. Datasets are constructed to simulate real-time updates of power system operating scenarios, which are characterized by continuous and repetitive variations. The fusion test set, which contains both the source and target domains, is used to compare stability assessment accuracy. The training data for datasets 1–4 correspond to datasets S0, T1, T2, and T3, which represent sequential changes. The test data form a fused dataset with learned scenarios. Detailed dataset settings are shown in Table 3.

**Table 3:** Sequential training and testing data structure for sustainable learning evaluation

Dataset	1	2	3	4
Train data	S0	T1	T2	T3
Test data	S0	S0+T1	S0+T1+T2	S0+T1+T2+T3

**Figure 5:**
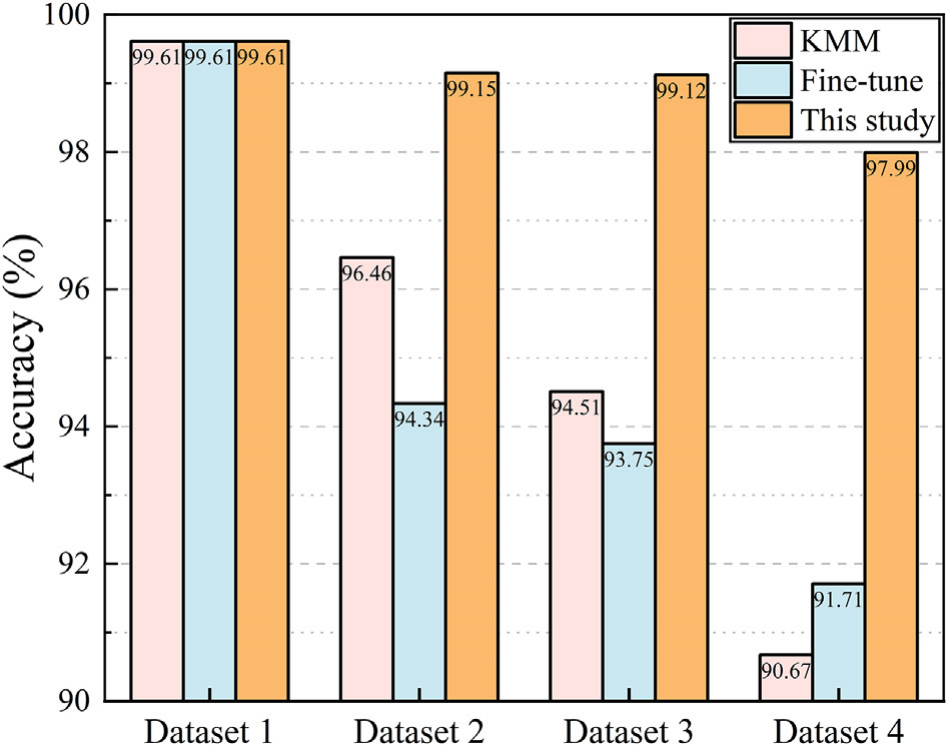
Sustainable learning ability comparison across sequential domain shifts

The test results, as illustrated in Figure 5, reveal that the fine-tuning method exhibits a continuous decline in performance on the learned system as the operating scenarios are updated, indicating that the model forgets acquired knowledge during the model updating process. This is attributed to the fine-tuning method’s lack of attention to the original operating scenario data and limited model structural parameters, resulting in poor performance on the learned system. The proposed method overcomes these limitations; it achieves non-forgetting updates and demonstrates strong sustainable learning capability in continuously varying scenarios.

The operating scenario may change continuously, so the TSA model may encounter some operating scenarios which have not been learned. Therefore, enhancing the model’s applicability to these unfamiliar scenarios can improve its assessment reliability in real-time power system applications. The proposed method considers the transient stability electrical characteristics in both the source and target domains. Considering there is an intersection in the feature space of different operating scenarios, the more operating scenarios the TSA model incorporates, the more likely it is to include unlearned operating scenarios and faults. Therefore, the proposed method, which uses data from multiple operating scenarios to train a shared feature extractor, can enhance the model’s generalization ability.

TSA model 1 is obtained through adversarial training using the dataset based on the original S0 and the target operating scenario T1. TSA model 2 and TSA model 3 are trained based on basic structure^7^^,^^8^^,^^9^ (as mentioned in the 2^nd^ paragraph of introduction) with the datasets from S0 and T1, respectively. The performance of the models is tested under four power system operating scenarios. Dataset 1 represents the test dataset in the labeled training data’s operating scenarios, while datasets 2 to 4 correspond to the test datasets in the other three operating scenarios. Detailed dataset settings are shown in Table 4.

**Table 4:** Training and testing datasets for evaluating TSA model generalization across multiple operating scenarios

Model^a^	Training data	Test dataset			
		Dataset1	Dataset2	Datase3	Dataset4
TSA model 1	S0	S0	T2	T1	T3
TSA model 2	S0	S0	T2	T1	T3
TSA model 3	T1	T1	T2	S0	T3

a TSA model 3 is trained based on operational scenario T1, but the other TSA models use S0 as their original scenarios. Therefore, when testing the models in different scenarios, TSA model 3 is evaluated using the T1 scenario as its original scenario and S0 as the unlearned scenario.

**Figure 6:**
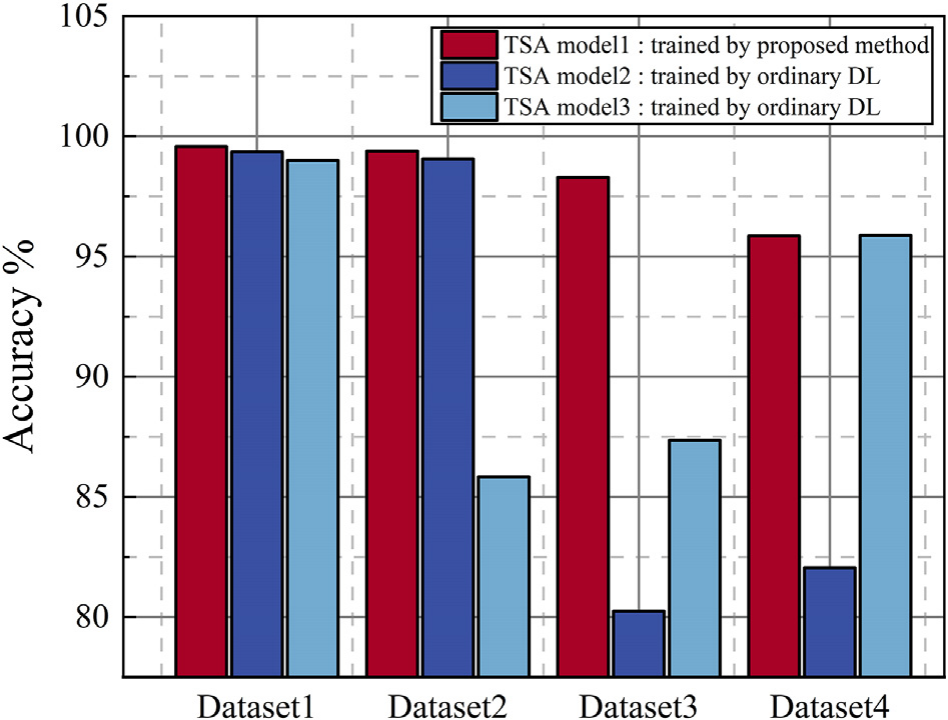
Generalizability comparison: proposed adversarial method vs. basic structure across operating scenarios

The test results are shown in Figure 6. TSA models 1, 2, and 3 achieve prediction accuracies of 99.57%, 99.35%, and 99.02%, respectively, on the operating scenarios used for model training, all surpassing 99%. This demonstrates the effectiveness of the TSA models based on the proposed architecture. Model 1, trained through adversarial training, achieves an accuracy of 98.29% on operating scenario T1, validating the feasibility of utilizing TSA information from the source domain to facilitate accurate predictions in the target domain. Furthermore, TSA model 1, trained using the proposed method, achieves the highest accuracy when evaluated on unlearned new systems T2 and T3, with accuracies of 99.38% and 95.85%, respectively, significantly outperforming TSA models 2 and 3 trained based on basic structure^7^^,^^8^^,^^9^ (as mentioned in the 2^nd^ paragraph of introduction). This confirms that the TSA model trained using the proposed method maintains high performance in unseen operating scenarios and fault scenarios, demonstrating excellent generalization capability.

During power system operations, phasor measurement unit (PMU) failures can result in the loss of transient stability data. The simultaneous occurrence of PMU data loss and changing operating scenarios can pose significant challenges. The following tests the ability of the proposed TSA model under unlearned scenarios with missing data.

The proposed method trains a shared feature extractor while aligning the source domain and target domain data, enabling the use of deep feature extraction networks to replace traditional missing data recognition and completion. As a result, the model can still achieve relatively accurate assessment when the target domain data are missing.

To create a robustness test dataset, 7.5%, 15%, and 30% of the data in the target domain sample set are randomly dropped and replaced by a default value “0”. This is to simulate real power system operating scenarios where data retrieval results may be contaminated due to PMU failures, communication congestion, and other factors. The results of the model on the robustness test dataset are shown in Tables 5 and 6. Sample transfer methods like KMM, model transfer methods like fine-tuning, and traditional feature transfer method each have certain limitations and are no longer applicable to data missing after changes in system operating scenarios. The test results indicate that TSA models based on basic structure^7^^,^^8^^,^^9^ (as mentioned in the 2nd paragraph of introduction) struggle to adapt to the simultaneous challenges of changing power system operating scenarios and data loss. The proposed adversarial training method, which uses a shared feature extraction network, can effectively function even in the absence of target domain data. When the proposed method encounters a data loss of up to 30% in new operating scenarios, it still achieves reliable TSA with an accuracy of over 96%.

**Table 5:** TSA method accuracy comparison with varying levels of missing target domain data

Method		Accuracy on target domain with missing data		
		7.5% missing	15% missing	30% missing
Without transfer learning		×	×	×
Transfer learning-based	KMM^a^	×	×	×
	Fine-tune	×	×	×
	Traditional feature-based transfer learning	×	×	×
	Proposed method	96.70%	96.43%	96.01%

a Kernel mean matching.

**Table 6:** Performance metrics of proposed TSA model with missing target domain data

Data	Evaluation metrics		
	Accuracy/%	Missing rate/%	False alarm rate/%
7.5% missing	96.70	6.79	0.20
15% missing	96.43	6.49	1.00
30% missing	96.01	4.57	3.61

### Large system test on a 2179-node province-level power system

In this section, analyses are conducted on a province level power grid system with 2,179 nodes located in Northeast China that serves over 100 million people. It consists of 2,970 AC transmission lines and 197 operational generators. Among these, there are 273 AC transmission lines operating at 500 kV and a total of 187 generators operating at 200 kV and 500 kV. The system setups are explained in the following text:

Source system S0: The load level is set to increase in 5% increments between 90% and 110%, resulting in five different load conditions. For each load condition, severe three-phase short circuit faults are placed at the 2% and 98% positions of all transmission lines. The faults start at 1.0 s and have a duration ranging from 0.1 s to 0.3 s with increments of 0.1 s. Time-domain simulations are conducted using PSASP software with a simulation step size of 0.01 s and a duration of 5 s. Successful simulations yield 8,126 transient stability samples, including 3,350 stable samples and 4,776 unstable samples.

Target power system T0: Five units are removed from the provincial-level power system of the source domain, including four 200 kV and 500 kV generators, and the system load is adjusted for balance. The same fault setting method as the source domain system is used. Successful simulations yield 8,123 transient stability samples, including 3,332 stable samples and 4,791 unstable samples.

The test results are shown in Table 7. Comparing the evaluation results of different network architecture feature extractors in source domain S0 demonstrates that the proposed serial-parallel architecture outperforms the commonly used CNN and LSTM structures, indicating the effectiveness of the proposed feature extractor architecture. Furthermore, the accuracy of the updated model in target domain T1 significantly improves from 85.60% to 96.39%. Additionally, the shared feature extractor obtained after updating the model retains information from S0 and expands the scope of feature extraction, increasing the prediction accuracy from 97.97% to 98.28% in source domain S0. The results demonstrate that knowledge is successfully integrated from both the source and target domains through the proposed method. The TSA performance test on the province-level power system demonstrated the practical applicability of the proposed method in large-scale power systems.

**Table 7:** Performance comparison of TSA models across different architectures and domains

Model	Test data	Evaluation metrics		
		Accuracy/%	Missing rate/%	False alarm rate/%
This study	S0	97.97	1.92	2.18
CNN^a^	S0	97.72	1.96	2.74
LSTM^b^	S0	97.41	3.92	0.60
This study	T1	85.60	14.36	14.46
Transferred by proposed method	S0	98.28	1.75	1.67
	T1	96.39	3.62	3.92

a Kernel mean matching.

b Transfer component analysis.

### Time consumption

The time involved in the application of the proposed TSA model mainly consists of three parts: sample preparation, model training, and model application. The time consumptions of these phases are shown in Table 8 and the results are discussed as follows:

**Table 8:** Time consumptions of the proposed TSA model at different stages

System	Sample Preparation (Offline)	Model training (Offline)	Model Application (On-line)
39-nodes	0.26s/sample	148.56s	0.11ms
2719-nodes	1.20s/sample	179.52s	0.17ms

Sample preparation: TSA samples mainly come from historical operation data of the power system and simulation data derived from computational models. Here, we focus on the simulation data. For the IEEE 39-bus system, we generated 14,243 samples in approximately 1 h (averaging 0.26 s per sample), while the larger provincial 2179-bus system required about 2 h and 42 min to generate 8,126 samples (averaging 1.2 s per sample). These preparation times are acceptable given that samples can be generated in advance of deployment.

Model training: The model training of the proposed method is also conducted offline. Consider a practical scenario where the TSA model has been trained on n scenarios, and then suddenly encounters a scenario change in power system operation and TSA needed to be done for the n+1^th^ scenario. In this case, the model can be directly applied to the n+1^th^ scenario without updates. This is because the average accuracy of the instantaneous assessment for new operating scenarios using a pre-trained model is 97.62%. Therefore, the model can be trained offline using both simulated data and actual operational data gathered afterward. Testing shows that model training takes 148.56 s for the IEEE 39-bus system and 179.52 s for the 2179-bus system.

Model application: In terms of real-time model application, our approach achieves remarkably fast assessment speeds, processing evaluations in 0.11 ms for the IEEE 39-bus system and 0.17 ms for the 2179-bus provincial system. These speeds readily exceed the requirements for online applications in real-world power systems.

It should be noted that all the tests are performed on a personal laptop. With sufficient computing resources in grid dispatching centers, all processes can be significantly accelerated by orders of magnitude. Additionally, it can be observed that although data preparation time for the 2179-bus system is significantly longer than that of the 39-bus system, the time consumption for model training and model application stages is comparable between large and small systems. This indicates that the time required by the TSA model based on the proposed architecture is not significantly sensitive to power system scale, demonstrating strong potential for scalability.

### Conclusion

This paper proposes a robust and transferable adaptive TSA framework based on shared feature extraction of the power system. The shared feature extraction achieves data alignment (between pre- and post-operational scenario changes) and feature extraction simultaneously. This approach can address the challenges to adapt to scenario changes and handle missing data when measurement system failures. In the source domain, a high-accuracy TSA model is trained with sufficient labeled data. When operational scenarios change, the framework transfers data and model knowledge simultaneously from the original scenario. It adaptively measures the distribution differences in data before and after operational scenario changes, and effectively updates the model through adversarial training, reducing the labeled data quality and quantity requirement during TSA model updating, and improving the model’s generalizability and sustainable learning capabilities. Case studies on the IEEE 39-node system and a 2179-node province-level power grid are carried out, and the main findings are summarized as follows.

(1) The performance of the customized feature extractor surpasses that of traditional architectures, achieving accuracies of 99.35% and 97.97% on two tested cases in the source domain.
(2) It is illustrated that during the adversarial training process, data in source and target domains are successfully aligned, and the extracted features can effectively identify the most physically impactful electrical quantities and extract useful information. Besides, the dynamic adversarial factor follows scenario change and converges quickly.
(3) The proposed framework achieves comprehensive improvements in the generalizability, robustness, and sustainable learning capability of the TSA model when the operating scenario changes.
  a. Accuracies of 99.57% and 98.29% in original and new scenarios are reached, respectively. When faced with totally unlearned operating scenarios, the model’s accuracy only drops to 95.85%, whereas that of basic TSA structures drop to 80.25% and 85.82%, respectively.
  b. The target domain prediction accuracy maintains above 96% even with 30% data loss in new scenarios. In comparison, retraining, model transfer, and traditional feature transfer methods fail under these scenarios.
  c. When the operating scenario changes four times continuously, the proposed model’s accuracy remains at 97.99% on the mixed dataset of these four scenarios, higher than the 91.91% accuracy of the traditional transfer learning method.

### Limitations of the study

This study focuses on TSA state prediction, like other studies,^8^^,^^9^^,^^33^^,^^34^^,^^35^^,^^36^ so predicting stability margins, which is also essential to real-world application, is beyond the scope of this study. However, in addition to predicting transient stability, our framework could also offer meaningful indicators of stability degree that supports the decision-making of system operators. When unexpected incidents occur, rapid response is essential. Since accurate physics-based simulation calculations are time-consuming, system operators must rely on simplified calculations or operation strategy tables to issue instructions. Given this limited understanding of system transient stability information, these instructions are often overly conservative (such as unnecessarily cutting off loads) to ensure grid security and stability. In such scenarios, the continuous output range (0–1) derived from the 2nd last layer of our model can serve as a valuable indicator for system stability. Values closer to 0 suggest a higher likelihood of instability, while those approaching 1 indicate greater system stability. When the system stability indicator is close to 1, system operators can be confident that the system will not lose stability or that only moderate response measures are needed. As such, our proposed method can serve as an effective reference to optimize decision-making, reducing unnecessary economic losses while ensuring safe and stable grid operation.

Only moderate modifications are required for the current framework to achieve stability margin predictions, including adjusting our training data labeling system to incorporate stability margins and modifying the model’s output. These improvements would enable our model to provide stability margins while maintaining its current classification accuracy. Our future studies will explore these improvements. Moreover, future work could further investigate integrating TSA with safety control strategies to enhance its practical value.

## Resource availability

The power system used for analysis includes the open-source IEEE 10-generator 39-nodes standard power system and the confidential 2179-node province-level power system of Northeast China. The data used for training and testing have been deposited at Zenodo and the original code developed has been deposited at GitHub.

### Lead contact

Requests for further information and resources should be directed to and will be fulfilled by the lead contact, Wei Ai (weiai@umich.edu).

### Materials availability

This study did not generate new unique reagents.

### Data and code availability

• The data used for training and testing TSA model have been deposited at Zenodo and are publicly available as of the date of publication at https://doi.org/10.5281/zenodo.14940971.
• This paper uses existing, publicly available data for IEEE 10-generator 39-nodes standard power system, accessible at https://doi.org/10.21227/cc5n-jb08.
• All original code has been deposited at GitHub and is publicly available at https://github.com/wdmzshjx/Adaptive-TSA-Method as of the date of publication.
• Any additional information required to reanalyze the data reported in this paper is available from the lead contact upon request.

## Acknowledgments

We would like to express our thanks for the support from the support of the 10.13039/501100001809National Natural Science Foundation of China (U1866602).

## Author contributions

Conceptualization, J.H., L.Z., W.A., and Y.L.; methodology, J.H. and L.Z.; software, J.H.; validation, J.H., L.Z., and J.L.; formal analysis, J.H. and W.A.; investigation, J.H. and X.C.; resources, Y.L., J.L., and X.C.; data curation, J.H.; writing – original draft, J.H., W.A., Y.L., and J.L.; writing – review and editing, J.H., W.A., Y.L., and J.L.; visualization, J.H. and W.A.; supervision, L.Z., W.A., and Y.L.; project administration, Y.L. and X.C.; funding acquisition, Y.L., J.L., and X.C.

## Declaration of interests

The authors declare no competing interests.

## STAR★Methods

### Key resources table

**Table:** 

REAGENT or RESOURCE	SOURCE	IDENTIFIER
**Deposited data**		

Data used for training and testing TSA model	This paper	https://doi.org/10.5281/zenodo.14940971
IEEE 10-generator 39-nodes standard power system	Li^24^	https://doi.org/10.21227/cc5n-jb08

**Software and algorithms**		

Adaptive TSA method	This paper	https://github.com/wdmzshjx/Adaptive-TSA-Method

### Method details

#### Framework for shared feature extraction

**Figure 7:**
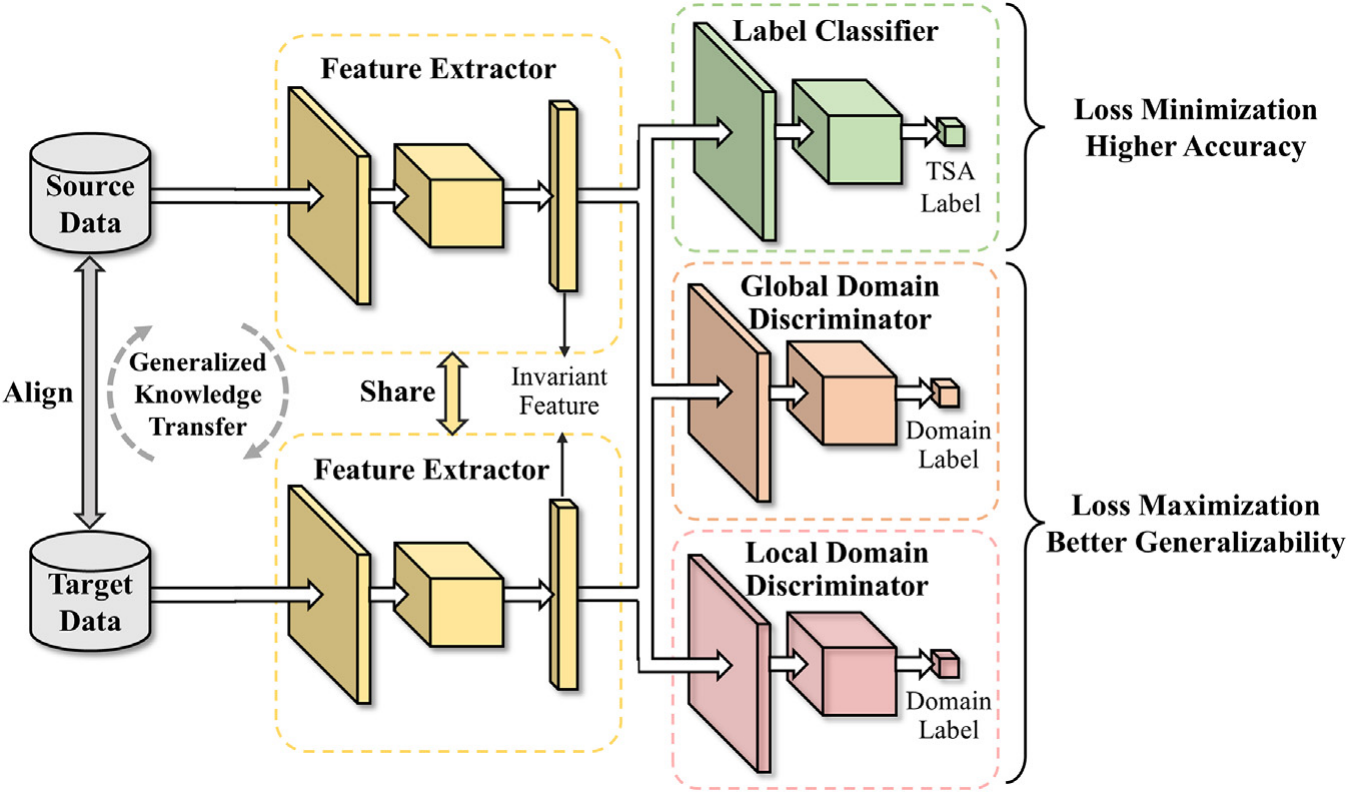
Training framework for the shared feature extractor

The proposed framework extracts shared features related to system stability that remain invariant before and after variations in operating scenarios. The transient stability of the system is assessed using these shared features. The training framework for the extractor is illustrated in Figure 7, which consists of three parts.

(1) The feature extractor *G*_*f*_ (with parameters *θ*_*f*_) aims to extract domain-invariant features that can be effectively transferred across the power system. *G*_*f*_ processes power system measurement data from both the source and target domains and aligns them to become domain-invariant, making it hard to differentiate between the source and target domains for the domain discriminator. These features are reliable for prediction, enabling the label classifier to accurately classify the features as stable (1) or unstable (0).
(2) The label classifier *G*_*y*_ (with parameters *θ*_*y*_) receives the extracted features and assesses the stability of the power system. It primarily aims to achieve precise stability classification of the high-dimensional features extracted by *G*_*y*_. The loss function of the label classifier is:

(Equation 1) $$L_{y}=-\frac{1}{n_{s}}\sum_{x_{i}\in D_{s}}Ly\left(G_{y}\left(G_{f}\left(x_{i}\right)\right),y_{i}\right)$$

Here, $L_{y}$ denote the label classifier loss function, $D_{s}={\left\{\left(x_{i}^{s},y_{i}^{s}\right)\right\}}_{i=1}^{n_{s}}$ represent *n*_*s*_ labeled examples in source domain.

(3) The domain discriminator consists of a global domain discriminator *G*_*d*_ (with parameters *θ*_*d*_) and local domain discriminators $G_{d}^{0}$ and $G_{d}^{1}$ (with parameters $\theta_{d}^{0}$ and $\theta_{d}^{1}$). The global domain and local domain discriminator align the source domain and target domain data from the perspectives of the global distribution and the local distribution of stable and unstable data, respectively. The importance of domain adaptation with respect to global distribution and local distribution will be introduced in the subsequent sections. The loss functions of the global domain discriminator and the loss function of the local domain discriminator are:

(Equation 2) $$L_{d}^{g}=\frac{1}{n_{s}+n_{t}}\sum_{x_{i}\in D_{s}\cup D_{t}}L_{d}^{g}\left(G_{d}\left(G_{f}\left(x_{i}\right)\right),d_{i}\right)$$

(Equation 3) $$L_{d}^{l}=\frac{1}{n_{s}+n_{t}}\left(\sum_{x_{i}\in D_{s}\cup D_{t}}L_{d}^{l0}\left(G_{d}^{0}\left({\hat{y}}_{i}^{0}G_{f}\left(x_{i}\right)\right),d_{i}\right)+\sum_{x_{i}\in D_{s}\cup D_{t}}L_{d}^{l1}\left(G_{d}^{1}\left({\hat{y}}_{i}^{1}G_{f}\left(x_{i}\right)\right),d_{i}\right)\right)$$

Here, ${\hat{y}}_{i}^{0}$ and ${\hat{y}}_{i}^{1}$ represent the predicted probability distribution over the stable and unstable classes for the input sample $x_{i}$, respectively. $D_{t}={\left\{x_{j}^{t}\right\}}_{j=1}^{n_{t}}$ represent *n*_*t*_ unlabeled examples in target domain. $d_{i}$ is the domain label, with 0 for source domain and 1 for target domain. $L_{d}^{g}$ is the global domain discriminator loss function, $L_{d}^{l}$ is the local domain discriminator loss, $L_{d}^{l0}$ is the local domain discriminator loss (unstable) function, $L_{d}^{l1}$ is the local domain discriminator loss (stable) function.

The operating scenarios of the power system are highly variable, and the differences in their distributions need to be dynamically and quickly measured. Traditional measure methods such as repetitive guessing or averaging multiple searches require a significant amount of time, which clearly does not meet the requirements for rapid assessment in the power system. Using feature extractors to extract deep features from both source and target domain data can leverage deep neural networks to enhance the accuracy and robustness of the measurement. Based on the A-distance measurement method,^37^ the losses of the domain discriminators are utilized for adaptive fine-tuning, eliminating the need for an additional classifier to calculate distances for updates. The global distribution discrepancy is measured based on the loss of the global domain discriminator, while the local distribution discrepancy is measured based on the losses of two types of local domain discriminators: the one for stable data and the other for unstable data. The adaptive weighting factors for distance calculation are then computed based on these measurements. The method is as follows:

(Equation 4) $$\begin{array}{l} d_{A,g}\left(D_{s},D_{t}\right)=2\left(1-2\left(L_{g}\right)\right)\\ d_{A,l}\left(D_{s}^{0},D_{t}^{0}\right)=2\left(1-2\left(L_{d}^{l0}\right)\right)\\ d_{A,l}\left(D_{s}^{1},D_{t}^{1}\right)=2\left(1-2\left(L_{d}^{l1}\right)\right)\\ \hat{\omega }=\frac{d_{A,g}\left(D_{s},D_{t}\right)}{d_{A,g}\left(D_{s},D_{t}\right)+\frac{1}{2}\left(d_{A,l}\left(D_{s}^{0},D_{t}^{0}\right)+d_{A,l}\left(D_{s}^{1},D_{t}^{1}\right)\right)} \end{array}$$

Here, $d_{A,g}\left(\cdot \right)$ represent global distribution discrepancy, $d_{A,l}\left(\cdot \right)$ represent global distribution discrepancy, $D_{s}^{0}$ and $D_{t}^{0}$ denote unstable samples, $D_{s}^{1}$ and $D_{t}^{1}$ denote stable samples. $\hat{\omega }$ is the dynamic adversarial factor.

#### Feature extractor design

Power system data primarily exhibits the following two characteristics.

(1) Feature Correlation: The variations in key electrical quantities in the power system are interrelated and coupled. Correlations exist among the generators data, such as power angle, voltage, power, frequency, and other electrical quantities.
(2) Time Series Characteristics: The power system is time-varying, and the measurements obtained from phasor measurement units are in the form of time series data.

**Figure 8:**
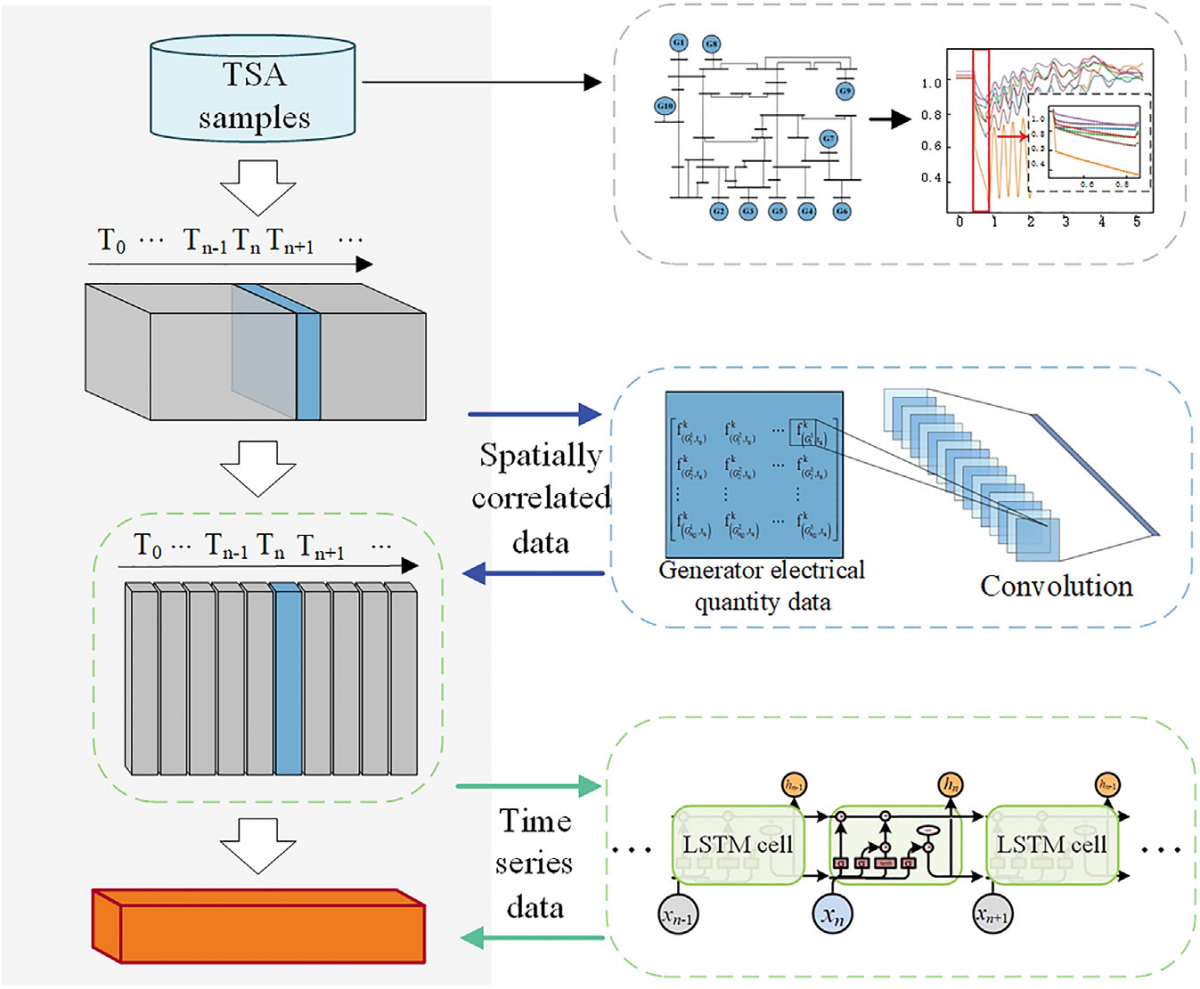
Diagram of power grid data feature extraction

Accounting for these characteristics, a CNN feature extraction network and an LSTM feature extraction network are employed in this paper to construct a series-parallel CNN-LSTM feature extraction network for power system data, as shown in Figure 8. The feature extraction process consists of two steps. First, to address the correlation patterns present in power system data, the CNN network is deployed to process the electrical quantities of each generator at every time segment. Utilizing local receptive fields allows the CNN network to adapt to the strong correlation and similarity within the data, enabling neurons to extract local features relevant to the power system $V_{t_{n}}^{CNN}$. Subsequently, considering the time-varying characteristics of the power system, the correlation features $V_{t_{n}}^{CNN}$ extracted from each time segment through the CNN network are transformed back into time series data $\left\{V_{t_{0}}^{CNN},V_{t_{1}}^{CNN},V_{t_{2}}^{CNN},...\right\}$. Finally, LSTM is utilized to exploit its capabilities in processing sequential data, and the ultimate power system features $V_{system}^{LSTM}$ are extracted.

To capture both spatial correlations and temporal dependencies in power system data, a two-stage power system feature extractor is designed. The structure and parameters are designed based on hyperparameter optimization, manual tuning, and drawing from successful frameworks in,^12^^,^^38^^,^^39^ as shown in Table 9.

**Table 9:** Structure and parameter design of the feature extractor

	Parameters setting
Input	([10,5] ∗ 9)
CNN^a^ feature extraction branch	Convolution(16, 2, ‘relu', get_same_padding)
	BatchNormalization(16)
	ReLU(16)
Intermediate output	([9, 800])
LSTM^b^ feature extraction branch	LSTM(800, 32, 2)
	LSTM(64, 32, 2)
	LSTM(50, 32, 2)
Output	([1, 288])

a Convolutional neural network.

b Long short-term memory.

#### Adversarial training strategy

**Figure 9:**
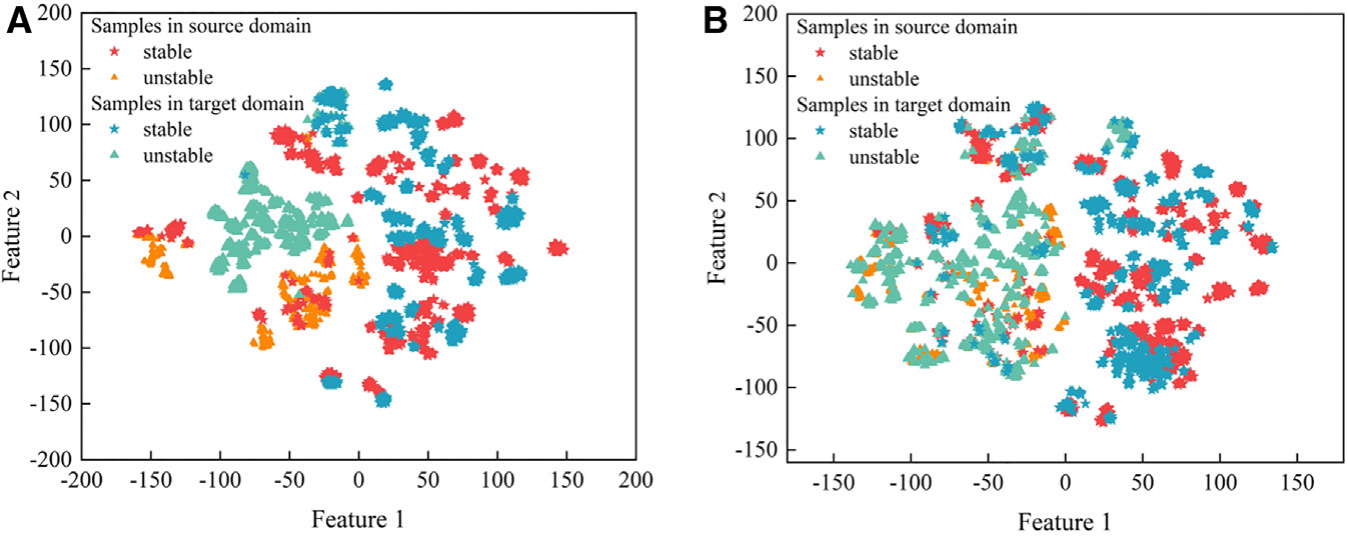
Feature space distribution shift after two system changes The feature space distribution between source and target domain exhibits substantial divergence when (A) removing one generator from the system, yet maintains considerable similarity when (B) disconnecting two transmission lines.

As the power system scenarios change, the data in the target domain (representing altered operating scenarios) and the source domain (representing original operating scenarios with sufficient samples) deviate from the assumption that the training and testing data are identically distributed, which underlies TSA with basic structure (as mentioned in the 2nd paragraph of introduction). Figure 9 presents the data distribution before and after changes in the power system operating scenarios, such as the removal of a generator and maintenance of two transmission lines. The accuracy of the TSA model, trained under the original operating scenario (using IEEE 39-node standard operating scenarios as the baseline in this paper) without transfer learning updates, decreased from 99.35% under the original scenarios to 81.97% and 82.21% under the new scenarios.

When significant changes (e.g., disconnecting a generator) occur, the overall distributions of the source and target domains differ, underscoring the importance of global distribution disparity. Conversely, when minor changes (e.g., disconnecting two transmission lines) occur, the overall data distributions in the source and target domains are highly similar. Thus, when assessing the power system’s operating state in these cases, the focus should be shifted to the local distribution differences, especially within stable and unstable samples. Besides, the contributions of the global and local data distributions change dynamically. To assess the significance of these two distributions in real-world scenarios, a dynamic adversarial factor (*ω*) is introduced to dynamically evaluate the importance of the global and local data distributions.

During the training process, the feature extractor *G*_*f*_ participates in adversarial training with the domain discriminators *G*_*d*_, $G_{d}^{0}$, and $G_{d}^{1}$. This adversarial training, based on domain adversarial alignment network,^19^ aims to unify feature processing and extract shared features with relevant to discriminating stable and unstable data states.

In order to fully leverage high-quality labeled data from the source domain to supplement the rough unlabeled data from the target domain, the optimization objective of the training process is to accurately distinguish high-quality labeled (source domain) samples and align the global and local distributions of data between the source and target domains, achieving model generalization. The overall objective of the training optimization process can be represented as:

(Equation 5) $$L\left(\theta_{f},\theta_{y},\theta_{d},{\theta_{d}^{c}|}_{c=1}^{C}\right)=L_{y}-\lambda \left(\left(1-\omega \right)L_{g}+\omega L_{l}\right)$$

Here, *λ* is an adjustable parameter.

During the actual training process, first, the parameters *θ*_*f*_ and *θ*_*y*_ of the feature extractor *G*_*f*_ and the classifier *G*_*y*_ are optimized by minimizing the loss of the label classification after feature extraction. Then, the optimization is carried out by maximizing the loss of the domain discriminator to optimize the parameters *θ*_*d*_, $\theta_{d}^{0}$, and $\theta_{d}^{1}$ of *G*_*d*_, $G_{d}^{0}$, and $G_{d}^{1}$.Then, the optimization is carried out by maximizing the loss of the domain discriminator. The training objectives for each part are as follows:

(Equation 6) $$\left({\hat{\theta }}_{f},{\hat{\theta }}_{y}\right)=\underset{\theta_{f},\theta_{y}}{\mathrm{arg min}}\;E\left(\theta_{f},\theta_{y},\theta_{d},\theta_{d}^{0},\theta_{d}^{1}\right)$$

(Equation 7) $$\left({\hat{\theta }}_{d},{\hat{\theta }}_{d}^{0},{\hat{\theta }}_{d}^{1}\right)=\underset{\theta_{d},\theta_{d}^{0},\theta_{d}^{1}}{\mathrm{arg max}}\;E\left(\theta_{f},\theta_{y},\theta_{d},\theta_{d}^{0},\theta_{d}^{1}\right)$$

To effectively achieve the above objectives, during the model parameter updates, a gradient reversal layer is introduced in the process of error backpropagation. The gradient reversal layer multiplies the gradient of the feature extractor parameters by a negative unit, thereby changing the objective of the feature extractor to increase the losses of the global domain discriminator, local stable domain discriminator, and local unstable domain discriminator. Let $\theta =\left\{\theta_{f},\theta_{y},\theta_{d},\theta_{d}^{0},\theta_{d}^{1}\right\}$ represent the network parameters, the model parameter update process with the selected hyperparameter *μ* is as follows:

(Equation 8) $$\Delta_{\theta }=\frac{\Delta L_{y}}{\Delta \theta }-\lambda \frac{\Delta \left(\left(1-\omega \right)L_{g}+\omega L_{l}\right)}{\Delta \theta }$$

(Equation 9) $$\theta \leftarrow \theta -\mu \Delta_{\theta }$$

The validity proof of adversarial training can be found in Data S1.

#### Adaptive TSA algorithm

A TSA model is constructed to predict transient stability in power systems. The output includes two categories, stable and unstable, forming a binary classification problem. In this paper, "0″ is used to represent instability, and "1″ is used to represent stability.

Five key electrical quantities of the generator are selected as input features: the relative power angle, voltage magnitude, frequency deviation, active power, and reactive power. Each candidate feature encompasses 10 temporal segments: one segment is taken 0.1 s before the fault event, five segments are evenly distributed throughout the fault duration, and four segments are evenly distributed within 0.2 s after the fault clearance.

The criteria for determining stability and instability are whether the relative power angle separation between generators exceeds a certain limit after a fault occurrence. The transient stability index (*I*_*TS*_) is constructed based on the angles of each generator after the fault, as shown in the following equation:

(Equation 10) $$I_{TS}=360-\left|\Delta \delta_{\max}\right|$$

Here, $\left|\Delta \delta_{\max}\right|$ denotes the maximum absolute value of the relative power angle difference between any two generators in the system after the fault occurrence. If *I*_*TS*_ > 0, the system is considered transiently stable. If *I*_*TS*_ < 0, the system is considered transiently unstable.

Due to the inherent stability of power systems, transient stability datasets often have a higher number of stable samples than unstable samples, leading to sample imbalance. Fixed-ratio loss functions can address the imbalance problem in TSA for only a specific fixed system topology, and they are unable to adapt to variations in input data distribution and changes in the power system’s topology. To dynamically adjust the parameter values of the loss function based on the distribution of input data, an adaptive cross-entropy loss function is employed in this study, as follows:

(Equation 11) $$l\left(\hat{p},y\right)=\sum_{k=0}^{1}w^{\left(k\right)}y^{\left(k\right)}\log\;{\hat{p}}^{\left(k\right)}$$

Here, *y* represents the actual labels of the data, $\hat{p}$ represents the probability vector output of the network, and *w*^(*k*)^ is inversely proportional to the proportion of that class in the dataset.

As predicting unstable samples as stable poses a greater risk to the system than predicting stable samples as unstable, providing a comprehensive and accurate description of the model’s predictive performance is important. This involves not only focusing on the overall accuracy of the model’s predictions but also evaluating its performance specifically on unstable and stable samples. Three metrics, including accuracy, miss rate, and false alarm rate, are used in this study to assess the predictive performance of the model.

**Figure 10:**
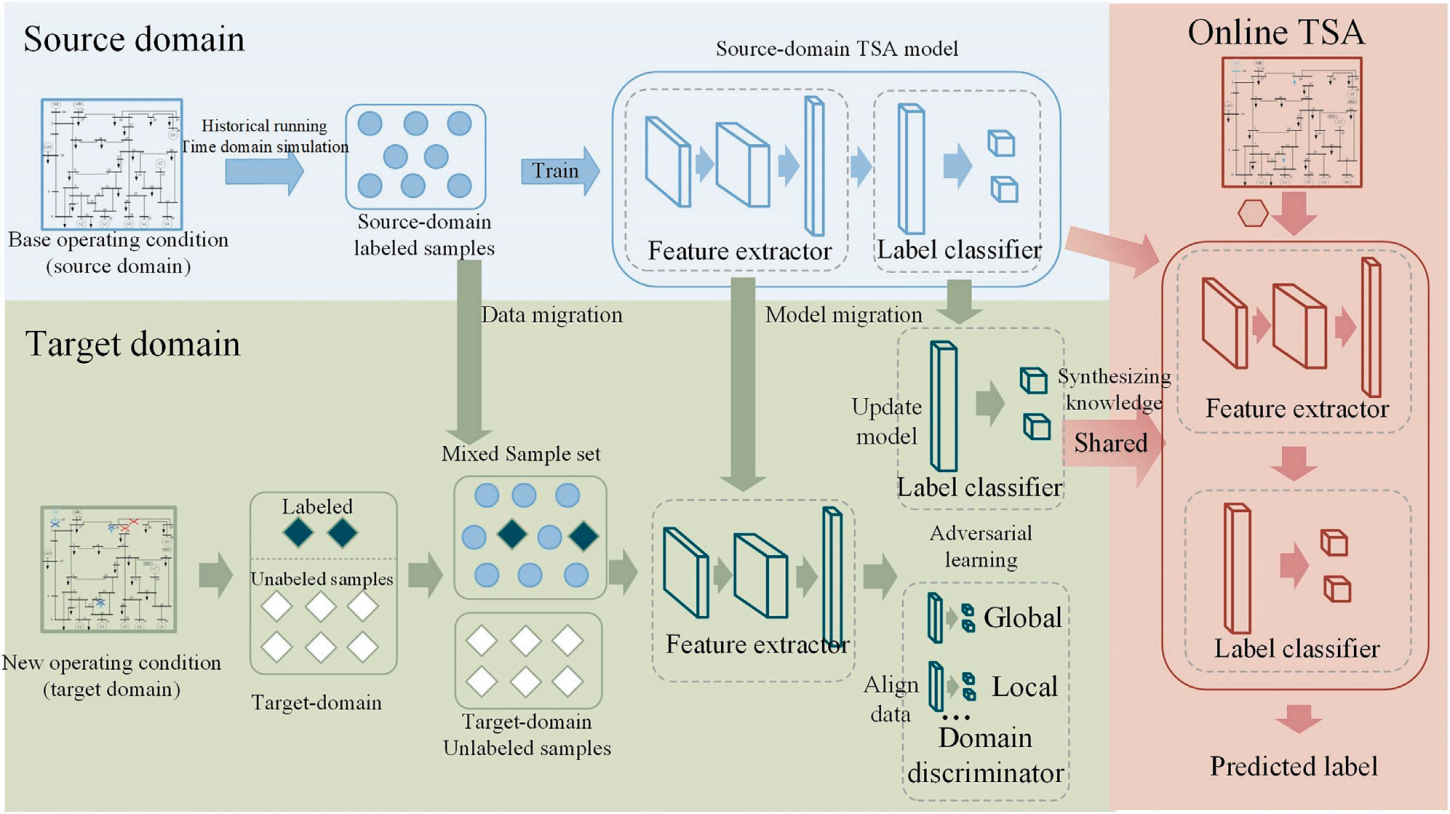
Framework of adaptive TSA

The proposed adaptive TSA framework, illustrated in Figure 10, consists of three main parts: source domain offline training, target domain transfer learning, and online transient stability assessment. (1) The source domain sample dataset is constructed based on historical operation data and time-domain simulation data. The dataset is then divided into training and testing sets, and sample weights (*w*^1^, *w*^0^) are determined. The forward neural network is optimized using the label classifier loss to train the source domain TSA model. (2) When the power grid operating scenario changes, the original TSA model no longer meets the assessment requirements. A large amount of unlabeled data from the target domain is obtained through short-term simulations, and a mixed dataset is created by combining the source domain training set with a small amount of labeled data from the target domain. The weight *ω* is adaptively measured to capture the global and local distribution differences between the source and target domains. A shared feature extractor is obtained by maximizing the domain classifier loss, in which the source and target domain data are aligned and the shared features are extracted. Also, the TSA model is updated by minimizing the label classifier loss. (3) Real-time online operation data are collected, and the well-trained model is employed to conduct rapid and accurate adaptive TSA.

### Quantification and statistical analysis

The sample generation settings are detailed in the results and discussion section, and the time consumption in Table 8 is calculated as the average of all testing results.

## Supplemental information

Data S1. Validity proof of adversarial training
