# Supplementary material for: An adaptive power system transient stability assessment method based on shared feature extraction
Source: iScience. 2025 Mar 6;28(4):112172. doi: 10.1016/j.isci.2025.112172 (PMC11987678; doi:10.1016/j.isci.2025.112172)
Supplement: Data S1. Validity proof of adversarial training [file mmc1.pdf]

iScience, Volume *28*

Supplemental information

An adaptive power system transient stability assessment method based on shared feature extraction

Jiexiang Hu, Le Zheng, Wei Ai, Yansong Li, Jun Liu, and Xinglei Chen

DATA S1. VALIDITY PROOF OF ADVERSARIAL TRAINING

The following provides mathematical proof that the TSA model trained using unlabeled data from the target power system and labeled data from the source domain, based on the methodology proposed in this paper, can make reliable predictions in the target domain [S1-S4].

TSA is a binary classification problem, with labels represented as 0 (unstable) and 1 (stable). The distribution of real labeled transient stability data is denoted as $\mathcal{D}=\{(x_i,y_i)\}_{i=1}^{n}$, and the true labeling function on this data distribution is represented by $f:\mathcal{X}\rightarrow[0,1]$. The classifier obtained through model training (i.e., the proposed TSA model) is denoted as $h:\mathcal{X}\rightarrow[0,1]$. Therefore, the error of the TSA model's predictions can be defined as follows:

$$\epsilon(h,f)=\mathbb{E}_{x\sim\mathcal{D}}[h(x)\neq f(x)]=\mathbb{E}_{x\sim\mathcal{D}}[|h(x)-f(x)|] \quad \text{(S1)}$$

The classification error of the TSA model *h* in the initial operating scenario (source domain) and the operating scenario after changes (target domain) can be respectively represented as:

$$\begin{aligned}\epsilon_{\mathrm{s}}(h)&=\epsilon_{\mathrm{s}}(h,f_{\mathrm{s}})\\ \epsilon_{\mathrm{t}}(h)&=\epsilon_{\mathrm{t}}(h,f_{\mathrm{t}})\end{aligned} \quad \text{(S2)}$$

Based on $\mathcal{H}\Delta\mathcal{H}$-distance theory, the prediction error of the target operating scenario can be bounded by the prediction error of the original operating scenario (source domain), the $\mathcal{H}\Delta\mathcal{H}$-distance between the source and target domains, and the shared error *C* under the ideal joint assumption. The upper bound of the constraint can be expressed as follows:

$$\epsilon_{\mathrm{t}}(h)\leq\epsilon_{\mathrm{s}}(h)+\frac{1}{2}d_{\mathcal{H}\Delta\mathcal{H}}(\mathcal{S},\mathcal{T})+C,\forall h\in H \quad \text{(S3)}$$

Here, *H* represents a hypothesis class, and *C* is a very small constant.

f represents the high-dimensional features extracted from the measured data of the power grid using the shared feature extractor G_f trained on both the source and target domain data. $\mathcal{H}$ represents the corresponding binary hypothesis class. D_d is obtained from the structure and corresponding parameters θ_d, θ^0_d, and θ^1_d of the domain adversarial networks G_d, G^0_d, and G^1_d proposed in this paper. The set of hypothesis classes is denoted as $\mathcal{H}_{\mathrm{Dd}}$. The domain adversarial networks are trained to distinguish the shared feature extractor from the high-dimensional features extracted from the measured data of the source and target domains. In the domain adversarial network model constructed in this paper, D_d has sufficient complexity to discriminate *f*, satisfying $\mathcal{H}\subseteq\mathcal{H}_{D_d}$.

$$\begin{aligned}d_{\mathcal{H}\Delta\mathcal{H}}(S,T)&=2\sup_{h_1,h_2\in\mathcal{H}}\left|\Pr_{\mathbf{f}\sim S}\left(h_1(\mathbf{f})\neq h_2(\mathbf{f})\right)-\Pr_{\mathbf{f}\sim T}\left(h_1(\mathbf{f})\neq h_2(\mathbf{f})\right)\right|\\ &=2\sup_{h\in\mathcal{H}\Delta\mathcal{H}}\left|\Pr_{\mathbf{f}\sim S}(h(\mathbf{f})=1)-\Pr_{\mathbf{f}\sim T}(h(\mathbf{f})=1)\right|\\ &\leq 2\sup_{h\in\mathcal{H}_{D_d}}\left|\Pr_{f\sim S}(h(\mathbf{f})=1)-\Pr_{\mathbf{f}\sim T}(h(\mathbf{f})=1)\right|\\ &=2\sup_{h\in\mathcal{H}_{D_d}}\left|\Pr_{\mathbf{f}\sim S}(h(\mathbf{f})=0)+\Pr_{\mathbf{f}\sim T}(h(\mathbf{f})=1)-1\right|\end{aligned} \quad \text{(S4)}$$

According to Eq. (13), the upper bound of $d_{\mathcal{H}\Delta\mathcal{H}}(S,T)$ can be obtained by finding a classifier $h(\cdot) \subseteq \mathcal{H}_{D_d}$ that maximally assigns the source domain data to 0 and the target domain data to 1. This is consistent with the training objective of the domain classifier, which can be obtained through the domain adversarial network and its parameters.

During the adversarial training process, as shown by the analysis of Eq. (12), the label classifier G_y iteratively aims to reduce the loss $\epsilon_s(h)$ in the original operating scenario. The feature extractor G_f extracts the shared features from the measured data of both the source and target domains. The domain adversarial networks G_d, $G^0{}_d$, and $G^1{}_d$ differentiate the extracted high-dimensional power grid features, approximating the upper bound of $d_{\mathcal{H}\Delta\mathcal{H}}(S,T)$. By jointly training the label classifier G_y, feature extractor G_f, and domain adversarial networks G_d, $G^0{}_d$, and $G^1{}_d$, reliable predictions can be achieved in the target domain.

SUPPLEMENTAL REFERENCES

[S1] Ben-David, S., Blitzer, J., Crammer, K., and Pereira, F. (2006). Analysis of representations for domain adaptation. Advances in neural information processing systems *19*.

[S2] Saito, K., Watanabe, K., Ushiku, Y., and Harada, T. (2018). Maximum classifier discrepancy for unsupervised domain adaptation. pp. 3723-3732.

[S3] Ben-David, S., Blitzer, J., Crammer, K., Kulesza, A., Pereira, F., and Vaughan, J.W. (2010). A theory of learning from different domains. Machine learning *79*, 151-175.

[S4] Zhao, H., Des Combes, R.T., Zhang, K., and Gordon, G. (2019). On learning invariant representations for domain adaptation. (PMLR), pp. 7523-7532.
